# Effects of Misalignment of *c*-axis on the Properties of Hydrogenation–Disproportionation–Desorption–Recombination Particles

**DOI:** 10.3390/ma17112635

**Published:** 2024-05-29

**Authors:** Xuhua Wang, Zilong Wang, Yuanfei Yang, Ningtao Quan, Zhongkai Wang, Haijun Peng, Hongbin Zhang, Xiaojun Sun, Shuai Cui, Dunbo Yu, Yang Luo

**Affiliations:** 1 China Grinm Group Co., Ltd., Beijing 100088, China; m13020075637@163.com (X.W.); wangzl@grirem.com (Z.W.); yangyf@grirem.com (Y.Y.); quannt@grirem.com (N.Q.); penghj@grirem.com (H.P.); 2 General Research Institute for Nonferrous Metals, National Engineering Research Center for Rare Earth, Grirem Advanced Materials Co., Ltd., Beijing 100088, China; wangzk@grirem.com (Z.W.); sunxj@grirem.com (X.S.); 3 Grirem High-Tech Co., Ltd., Beijing 100088, China; rosesbass@126.com (H.Z.); cs837041286@163.com (S.C.)

**Keywords:** HDDR Nd-Fe-B powders, permanent magnet, remanence, hysteresis behaviour

## Abstract

Hydrogenation–Disproportionation–Desorption–Recombination (HDDR) Nd_2_Fe_14_B particles have excellent magnetic properties, but the magnetic properties of powder are not uniform across different particle sizes. The remanence and maximum magnetic energy products of samples with a particle size of 120 μm are 14.0 kGs and 41.35 MGOe, while the products of samples with a particle size of 60 μm are only 13.3 kGs and 36.31 MGOe. The macroscopic morphology of HDDR Nd_2_Fe_14_B particles and the gradient distribution of microstructures in different micro-regions were observed. By modifying the macroscopic morphology of the particles, the poorly oriented clusters on the surface of the particles were precisely eliminated, and the remanence and maximum magnetic energy products of the particles increased to 14.5 kGs and 45 MGOe, respectively. Compared with the original particles, the samples after mechanical grinding had better grain arrangement. The effects of the nanocrystalline *c*-axis and field misalignment angle θ on the magnetic properties of HDDR Nd_2_Fe_14_B particles were investigated through micromagnetic simulation. The targeted removal of macroscopic defects on the particle surface contributed to a 3.6% increase in remanence and an 8.8% increase in the maximum magnetic energy product, offering a promising approach to enhance the microstructure of high-performance HDDR Nd_2_Fe_14_B particles.

## 1. Introduction

In recent years, clean energy technology has played an increasingly important role in addressing the environmental pollution caused by fossil fuels [1,2,3]. Devices made of permanent magnet materials are being developed with enhanced magnetic properties, reduced weight, smaller size, and higher temperature tolerance, serving as the cornerstone for progress in clean energy technology [4,5]. Nd_2_Fe_14_B magnetic powder, known for its excellent room temperature magnetic properties, is widely used in environmentally friendly household appliances [6,7]. However, as devices continue to integrate and miniaturize, the inconsistent magnetic characteristics of Nd_2_Fe_14_B powder are becoming evident as devices shrink, impeding their application in compact, micro, and irregular structures [2,8].

Various methods are used to prepare nanoscale Nd_2_Fe_14_B magnetic powder [9,10,11]. The HDDR process proposed by Takeshita and Nakayama stands out as a unique method that allows for the direct large-scale production of magnetic powder with ultrafine grains that match the size of magnetic domains [12]. The key to achieving high anisotropy in HDDR Nd_2_Fe_14_B lies in the complete inheritance of the orientation of coarse grains from the precursor alloy strip [13,14,15]. Current research primarily concentrates on enhancing the coercivity of NdFeB magnets by manipulating the material’s microstructural characteristics through controlling the size of the precursor and reaction processes [16,17,18,19]. The microstructural traits of nanocrystalline Nd_2_Fe_14_B materials are crucial for their functionality [3,20,21]. Adjusting and optimizing the microstructure of Nd_2_Fe_14_B nanocrystalline provides a sustainable and economical method to improve the overall magnetic properties of magnets [22,23,24,25,26]. 

It was observed in this study that after the HDDR reaction, there are differences in the microstructure of the magnetic particles, and the distribution of nanoscale clusters on the surface of the particles is uneven. The effect of grain structure gradient distribution in single particles on magnet properties was studied by simulation. The precise control of the internal microstructure of grains during the HDDR process is challenging due to the non-uniform and unstable reaction [27,28]. However, analyzing the relationship between the structure and magnetic properties of nano-polycrystalline magnetic powder through fine-tuning the micro-layered structure of particles is feasible [29]. Mechanical exfoliation can be used to grind powders to produce finer particles to adjust the micro-layered structure, controlling aspects like size, shape, and interface [30,31]. By controlling the separation of the peripheral and central regions of the particles, their corresponding relationship between the microstructure and magnetic properties of the HDDR magnetic powder particles can be obtained.

In summary, adjusting the gradient microstructure is a promising strategy to explore the impact of multi-layered structures on the anisotropy of magnetic powder, shedding light on the development of highly anisotropic HDDR powder.

## 2. Materials and Methods

HDDR Nd_2_Fe_14_B powder was prepared from homogeneous alloy ingot with a nominal composition of Nd_12.5_Fe_bal._B_6.4_Ga_0.3_Nb_0.2_ (at.%). The HDDR magnetic powder produced in the same batch is split into five groups denoted as P_A_, P_B_, P_C_, P_D_, and P_E_, based on particle size in the following ranges: 150–180 μm, 120–150 μm, 90–120 μm, 60–90 μm, and less than 60 μm, respectively (Grirem High-Tech Co., Ltd., Beijing, China). The particles were placed in s stainless steel cylinder, and the sharp edges of the surface of the particles were polished by the friction and impact between the particles. The particle size of the treated particles showed an obvious bimodal distribution. We defined the part of the treated particles with a diameter greater than 90 μm as the internal region of the particles. The treated particle diameter less than 60 μm was mainly the external region of the particle.

The magnetic properties of the powders were evaluated by a vibrating sample magnetometer (VSM, Quantum Design, San Diego, CA, USA) with a maximum pulse magnetic field of 3 T. P_A_, P_B_, P_C_, P_C_ internal region, P_C_ external region, P_D_ and P_E_ were used to form bonded magnets. The surface was treated with a 0.1 wt.% silane coupling agent, followed by the creation of a uniform compound made by mixing 1.5 wt.% epoxy resin with acetone as a solvent. Prior to curing, the mixture was exposed to a 2T magnetic field to obtain complete orientation, which was then analyzed using X-ray diffractometry (XRD). 

The morphology of HDDR particles was observed by a scanning electron microscope (SEM, Tescan Vega II, Brno, Czech). The morphology and orientation distributions of the Nd_2_Fe_14_B nanocrystalline within the particles of different regions were studied using a Transmission Electron Microscope (TEM, FEI Tecnai, Valley City, ND, USA). Samples were extracted using a precise focused ion beam (FIB) cutting technique and then carefully scanned layer by layer along the radial direction. 

The 5 × 5 × 5 units with a 30 nm side length and a saturation magnetization intensity (Js) of 1.61 T served as the primary phase of the Nd_2_Fe_14_B nanostructure. By changing the uniaxial anisotropy direction, the misalignment angle θ (where θ represents 0~45°) between the grain’s easy magnetization axis direction and the *z*-axis direction was simulated. The anisotropy constant (K1) was 4.5 MJ/m^3^, and the uniaxial anisotropy direction was (0, sin(θ), cos(θ)). The exchange stiffness (A) of Nd_2_Fe_14_B was set to 12.5 PJ/m. The grain boundary phase demonstrated ferromagnetism, with magnetic parameters of 0.15 T, 0 MJ/m^3^, and 5 PJ/m in order. If the grain boundary phase wetted the grain, the model’s thickness was set to 3 nm; otherwise, it was set to 0 nm.

The demagnetization curves for the following five scenarios were simulated:

i. One Nd_2_Fe_14_B grain with disorientation, located at the body center,
ii. One Nd_2_Fe_14_B grain with disorientation, situated at the face center,
iii. One Nd_2_Fe_14_B grain with disorientation, located at the face center, but with a non-wetted grain boundary in the *z*-axis direction,
iv. Twenty-five Nd_2_Fe_14_B grains with disorientation, located on the surface of a magnetic cluster,
v. All grains outside of the magnetic cluster had a misalignment angle θ with the orientation of the central grain.

## 3. Results

### 3.1. Composition and Properties of HDDR Powders

**Figure 1:**
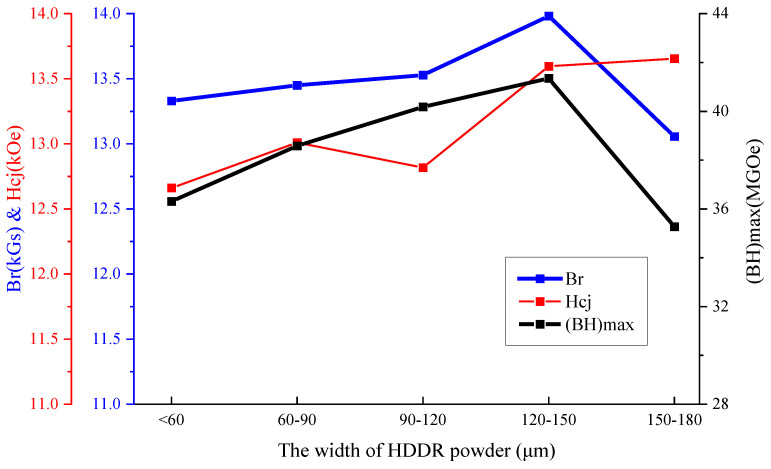
Relationship between magnetic properties of Br, H_cj_ and (BH)_max_ with particle size variation.

The magnetic properties were evaluated by VSM testing and the magnetic properties obtained are shown in Figure 1, while the test results are shown in Table 1. The magnetic properties of the original magnetic particles show an obvious particle size effect. The remanence and maximum magnetic energy products of magnetic particles increase first and then decrease with the increase in average particle size. When the particle size is in the range of 120~150 microns, the maximum values of the remanence, coercivity and maximum magnetic energy product reach 13.9 kGs, 13.6 kOe and 41.4 MGOe, respectively. 

**Figure 2:**
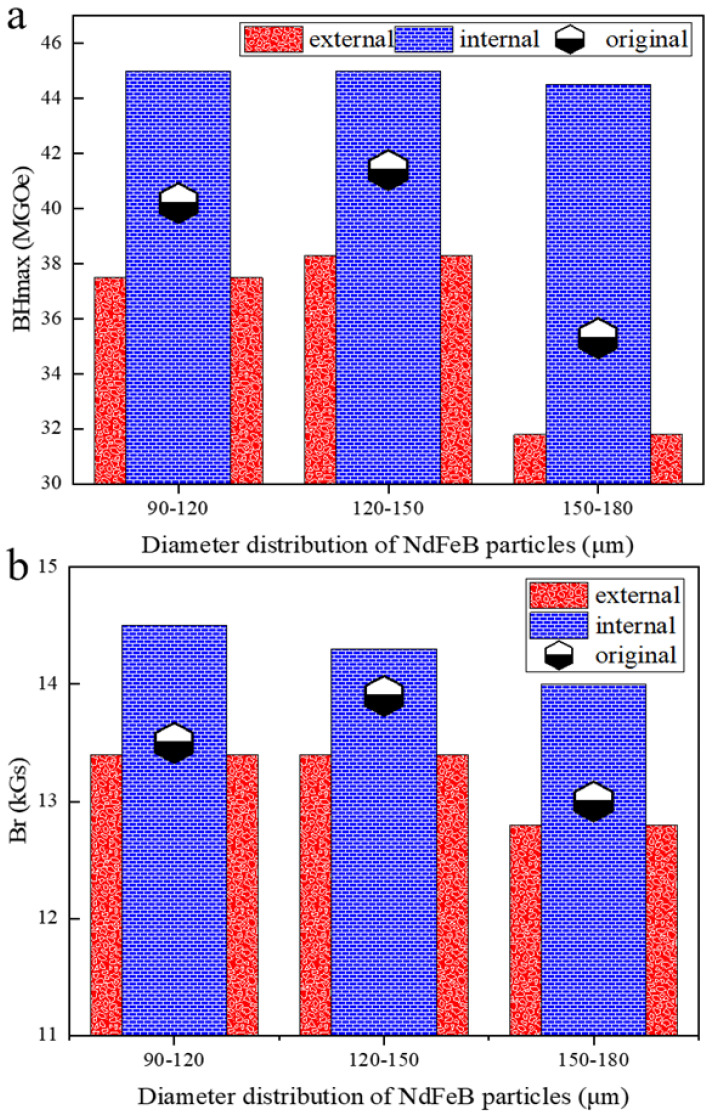
Difference of magnetic properties between internal and external regions of particles. (**a**) Maximum magnetic energy product (BH_max_); (**b**) saturation magnetization (M_s_).

After the magnetic particles are categorized by particle size to obtain magnetic particles with stable magnetic properties, the microstructure of the original magnetic particles was modified. Then, the comprehensive magnetic properties of the internal and external region of the P_A_, P_B_ and P_C_ were tested, as shown in Figure 2a,b. The remanence of the internal region of the powder was increased by 7.1%, 2.1% and 7.6%, respectively, and the maximum magnetic energy product was significantly increased by 11.9%, 8.7% and 26.2%, respectively. It is worth noting that after optimizing the magnetic particle structure, the remanent magnetic and the maximum magnetic energy product of each group of particles tend to be consistent. The magnetic properties of the sample representing the internal region of the particle are higher than those of the original powder. Although the magnetic properties of samples representing the external region of the particles are lower than those of the original particles, their magnetic properties are better than those of P_D_, and P_E_ directly produced by the HDDR method. 

Although there are great differences in the magnetic properties of P_A_, P_B_ and P_C_, there is no significant difference in the oxygen content of particles with diameters greater than 90 μm, which are all about 0.1 wt.%. After surface structure optimization, the oxygen content of the internal region of the particles is similar to that of initial magnetic particles, indicating that the particles were not significantly oxidized after being polished by the friction and impact. 

The oxygen content test showed that the oxygen content of P_D_ and P_E_ was high, which was close to 0.2 wt.%. The oxygen content of the external region was slightly lower than P_D_ and P_E_, about 0.15 wt.%. Although the oxygen content is similar, the magnetic properties of the magnetic particles are significantly different. The test results show that the degradation of HDDR magnetic powder is not only due to the oxidation of particles.

### 3.2. Microstructures of Particles

**Figure 3:**
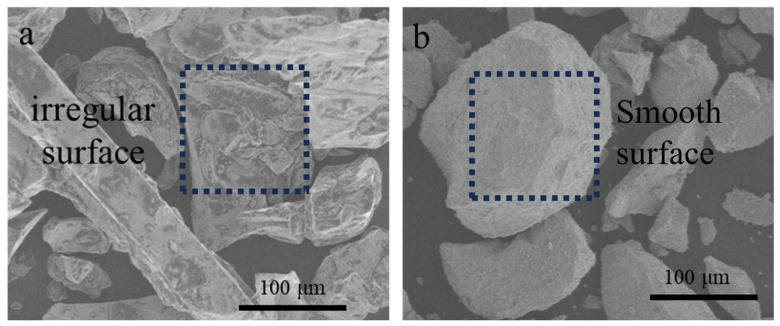
Morphology of powders by SEM. (**a**) Original powders; (**b**) the powders after modifying.

The surface morphology of HDDR powder before and after structure optimization was observed by scanning electron microscopy (SEM), as shown in Figure 3a,b. The surface of HDDR particles before modification is very rough, and irregular polyhedra and sharp edges can be observed. After grinding and collision, the surface of magnetic particles tends to be smooth, and the clusters attached to the abnormal surface morphology are separated. By grinding and colliding the original magnetic powder, the abnormal nanocrystals on the surface of the particles can be removed, and the irregular structure on the surface of the particles can be separated from the central region of the particles to optimize the internal microstructure of the magnetic powder particles.

**Figure 4:**
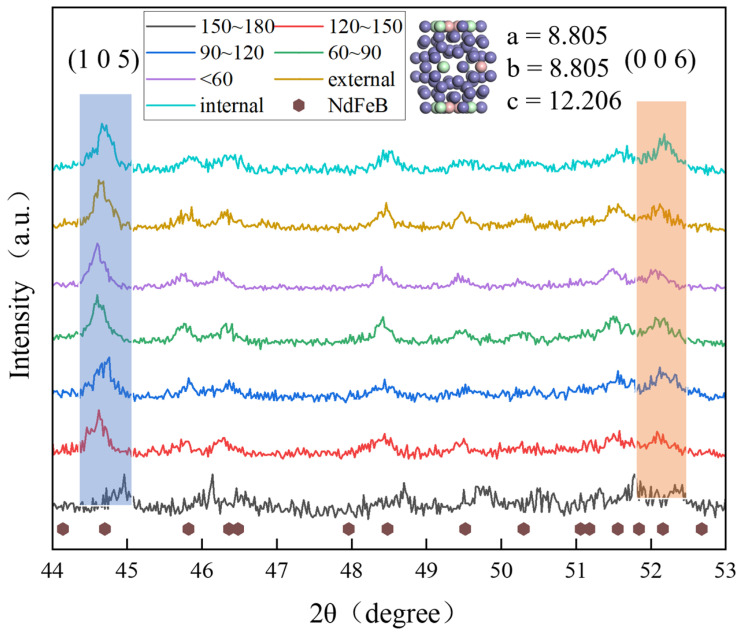
X-ray diffraction curves of magnetic powders.

The bonded magnet obtained after the orientation of HDDR magnetic powder is used for XRD testing. The XRD test results are shown in Figure 4, and the ratio of characteristic peaks is shown in Table 2. The intensity ratios of characteristic peak (0 0 6) and characteristic peak (1 0 5) of Nd_2_Fe_14_B were selected to characterize the preferred orientation of the grain inside the HDDR powder. The experimental results show that the intensity ratio of diffraction peaks changes with the change in particle size, and the change trend is the same as that of the maximum magnetic energy product, which also shows a trend of first increasing and then decreasing. After the structure optimization, the intensity ratio of the diffraction peak in the internal region of the particle is greatly improved, while that of the external region of the particles in the stripped region decreases significantly. The XRD results show that the nanocrystalline orientation of HDDR particles with different diameters is different. The degree of preferred orientation of nanocrystals within individual particles is different, and the *c*-axis orientation of grains located in the internal region of the particles is consistent.

SEM observation, XRD characterization and magnetic property tests all show that there are significant differences in the microstructure and magnetic properties of the nano-polycrystalline structures between the internal and external region of the particles. The standard diffraction peaks of the Nd_2_Fe_14_B phase can be seen from the standard PDF card. The surface structure spalling caused by mechanical exfoliation greatly optimizes the properties of magnetic particles and weakens the particle size effect caused by the difference in microstructure distribution. 

### 3.3. Microstructure of Nd_2_Fe_14_B Nanocrystals

**Figure 5:**
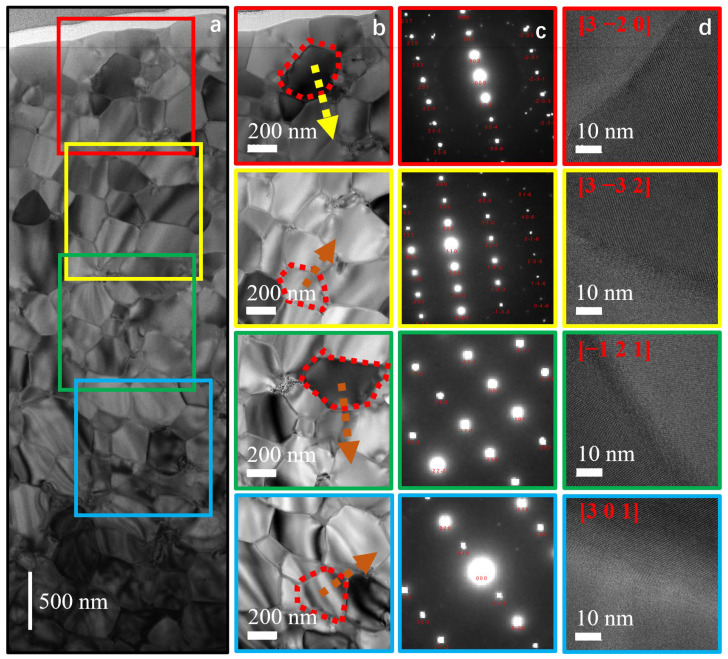
TEM image of external regions. (**a**) FIB samples extracted along the particle radial direction, (**b**) Nd_2_Fe_14_B nanocrystals, (**c**) selective area electron diffraction and (**d**) HRTEM of the grain boundary phase.

The representative microstructure of HDDR particles was selectively extracted from the external and internal region of HDDR particles by the FIB technique for layer-by-layer scanning. The test results of the external region of HDDR particles are shown in (a), (b), (c) and (d) in Figure 5, where (a) is the TEM image of the FIB sample extracted along the radial direction of the particle, (b) is the microscopic morphology of the local grain observed layer by layer on the FIB, and (c) is the selective area electron diffraction (SAED) image of the observation region, where the red number is the crystal band axis. The orange arrow is the projection of the *c*-axis orientation of the grain on the observed plane, while the yellow arrow is the *c*-axis orientation of the grain. The SAED test results show that the *c*-axis orientation of the grain on the external region of the grain is not parallel, and the calculated angle between the *c*-axis of the outer grain and the other three grain angles are 51°, 20° and 70°, respectively. There is no obvious correlation between the *c*-axis arrangement of grains. In addition, d is the high-resolution transmission electron microscopy (HRTEM) image of the grain in the selected region. The test results show that there are non-wetting grain boundaries between the surface grains and the lattice of adjacent grains in direct contact. In the process of grain growth, the non-wetting grain boundary will promote rapid abnormal grain growth and weaken the orientation relationship between the surface grains. In fact, it is observed that the *c*-axis orientation of grains in the edge region tends to be randomly distributed, and the *c*-axis misalignment angle between grains is greater than 20°.

**Figure 6:**
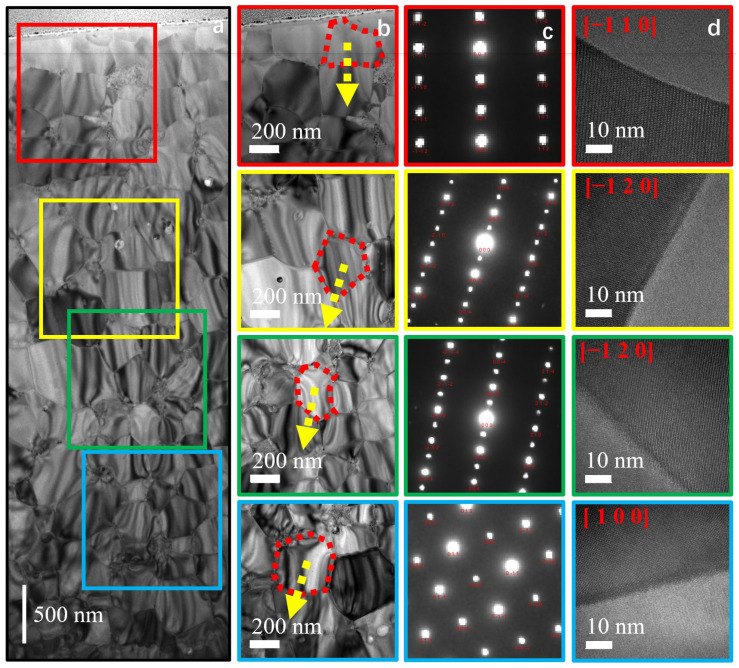
TEM image of external regions. (**a**) FIB samples extracted along the particle radial direction, (**b**) Nd_2_Fe_14_B nanocrystals, (**c**) selective area electron diffraction and (**d**) HRTEM of grain boundary phase.

The test results of the internal region of HDDR particles are shown in (a), (b), (c) and (d) in Figure 6, where (a) is the FIB sample extracted from the internal region of the particles, (b) is the microscopic morphology of the local grain observed layer by layer on the FIB, (c) is the SAED picture of the observation region, where the red number is the crystal band axis, and the yellow arrow is the direction of the *c*-axis of the grain. The results show that the *c*-axis of the grain in the central region of the particle is in the same plane, and the angles between the grain orientation indicated by the sample and the *c*-axis of the other three grains are 16°, 10° and 17°. The *c*-axis orientation of the grains in the central region is highly consistent, and the *c*-axis misalignment angle between grains is less than 20°. In addition, d is the HRTEM image of the grain in the central region. It can be seen that the nanocrystalline polycrystals are evenly coated with rare-earth-rich phase, and the average grain boundary width reaches 2~3 nm.

The results of the microstructure test show that the easy magnetization axes of HDDR particles are not completely parallel. In particular, the easy magnetization axes between the grains located in external region of the particles are not completely parallel, and the *c*-axis of the grains has no obvious correlation. In addition, the grains located in external regions are almost in direct contact, and the wettability of the rare-earth phase is very poor.

### 3.4. Micromagnetic Simulation in HDDR Powder

Based on the results of SEM and TEM, it can be found that the direction of the easy magnetization axis of HDDR NdFeB grains is not parallel. The change in magnetic properties of magnetic particles with the deterioration of the orientation degree was investigated by flipping the axes of easy magnetization at different positions and with different numbers of nanocrystals. The blue cube represents that the *c*-axis is parallel to the *z*-axis, while the red cube has misalignment between the *c*-axis the *z*-axis.

**Figure 7:**
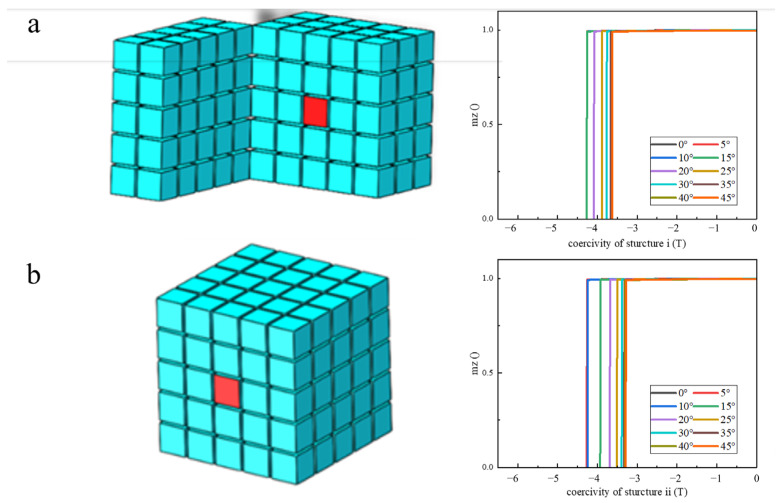
Models and demagnetization curve of orientation deviation of single grains. (**a**) Abnormal grains located in the center of structure i; (**b**) abnormal grains located in the center of structure ii.

Models i and ii shown in Figure 7a,b represent the effect of the direction of the easy magnetization axis of a single grain within a magnetic cluster on the magnetic properties. The locations of abnormal grains at the center or surface of magnetic clusters correspond to two structures, respectively. The deviation angle gradually increases from 0° to 45° to simulate the demagnetization curves of structures i and ii. The simulation results show that the coercivity of the model with the abnormal orientation grain located in the center is higher than that of the model with the abnormal orientation grain located on the surface. When the deviation angle of the abnormal oriented grains in the cluster is less than 10°, the coercivity has no obvious change. It shows that the coercivity will not decrease when the easy magnetization axis is only slightly misaligned. When the instability angle further increases to 20°, the coercivity decreases rapidly to about 80% of the initial value as the deviation angle between orientations continues to increase. 

**Figure 8:**
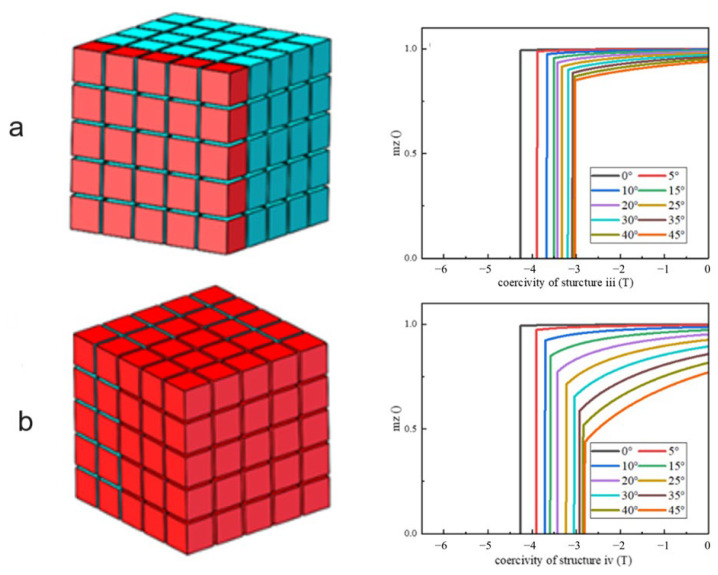
Models and demagnetization curve of orientation deviation of multiple grains. (**a**) Abnormal grains located in monolayer of structure iii; (**b**) Abnormal grains located on outer surfaces of structure iv.

Models iii and iv shown in Figure 8a,b represent the effect of the direction of the easy magnetization axis of multiple grains within the magnetic cluster on the magnetic properties. The single layer of abnormal grains on the surface of the cluster and all the outer surfaces of the cluster correspond to two structures, respectively. The deviation angle gradually increases from 0° to 45° to simulate the demagnetization curves of models iii and iv. The simulation results show that with the increase in the orientation deviation angle and the increase in the number of misaligned grains, not only the coercivity decreases, but also the maximum magnetic energy product and the remanence decrease significantly. In summary, the magnetic properties of magnetic particles will be significantly reduced when the internal grain orientation of the particles is not matched. In particular, the surface of magnetic particles is prone to misalignment of the easy magnetization axis, which is not conducive to the improvement of the magnetic properties of particles.

## 4. Discussion and Conclusions

Hydrogenation–Disproportionation–Desorption–Recombination (HDDR) Nd_2_Fe_14_B particles have excellent magnetic properties, but the magnetic properties of powder are not uniform across different particle sizes. The experimental results indicate that the distribution of grain microstructure inside the particle is not uniform. The interior of the particle exhibits a perfect uniaxial arrangement of the *c*-axis of the grains, while the periphery shows a random distribution.

Micromagnetic simulation was used to study the effects of the grain misalignment angle of the nanocrystalline *c*-axis on the magnetic properties of Nd_2_Fe_14_B powder. The simulation results show that coercivity and remanence decrease as the misalignment angle increases. Ensuring the uniformity of individual particles is the key to improving the magnetic properties of HDDR magnetic powder. The macroscopic morphology of HDDR Nd_2_Fe_14_B particles and the gradient distribution of microstructure in different micro-regions were observed. By modifying the macroscopic morphology of the particles, the poorly oriented clusters on the surface are precisely eliminated, leading to an increase in remanence and maximum magnetic energy products. A new method is proposed to improve the magnetic properties of HDDR Nd_2_Fe_14_B particles by removing the *c*-axis random distribution area.

In conclusion, the misalignment angle of the *c*-axis of HDDR magnetic particles significantly impacts their magnetic properties. By addressing this misalignment and ensuring uniformity at the particle level, the magnetic properties of HDDR magnetic powder can be improved. The targeted removal of macroscopic defects on the particle surface offers a promising approach to enhancing the microstructure of high-performance HDDR Nd_2_Fe_14_B particles, leading to increased remanence and maximum magnetic energy products. 

## Figures and Tables

**Table 1:** Magnetic properties of magnetic powders.

Diameter of Particles	Position of Particles	*B*_r_	ΔB_r_	*H*_cj_	(*BH*)max	Δ(*BH*)max	Oxygen Content
		kGs	%	kOe	kJ/m^3^	%	wt.%
P_E_	Whole	13.2	-	11.3	31.5	-	0.26
P_D_	Whole	13.3	-	12.7	36.3	-	0.18
	Whole	13.5	-	13.6	40.2	-	0.10
P_C_	Internal	14.5	7.4	12.5	45.0	11.9	0.10
	External	13.9	3	12.8	41.3	−2.7	0.15
	Whole	14.0	-	13.6	41.4	-	0.08
P_B_	Internal	14.3	6.7	12.6	45.0	8.7	0.08
	External	13.4	0	12.3	38.3	−7.5	0.14
	Whole	13.0	-	13.7	35.3	-	0.09
P_A_	Internal	14.0	7.7	12.6	44.5	26	0.09
	External	12.8	−1.5	11.6	31.8	−9.9	0.15

**Table 2:** Intensity of XRD characteristic peaks.

	150~180	120~150	90~120	60~90	<60	Internal	External
(0 0 6)	19	55	55	54	57	60	56
(1 0 5)	27	75	69	92	123	74	96
I_(0 0 6)_/I_(1 0 5)_	70.4%	73.3%	79.7%	58.7%	46.3%	81.1%	58.3%

## Data Availability

Data are contained within the article.
